# Light-Driven Rotation and Pitch Tuning of Self-Organized Cholesteric Gratings Formed in a Semi-Free Film

**DOI:** 10.3390/polym9070295

**Published:** 2017-07-21

**Authors:** Ling-Ling Ma, Wei Duan, Ming-Jie Tang, Lu-Jian Chen, Xiao Liang, Yan-Qing Lu, Wei Hu

**Affiliations:** 1 National Laboratory of Solid State Microstructures, Collaborative Innovation Center of Advanced Microstructures and College of Engineering and Applied Sciences, Nanjing University, Nanjing 210093, China; malingling9150@foxmail.com (L.-L.M.); wwtutu@163.com (W.D.); mjtang0330@foxmail.com (M.-J.T.); yqlu@nju.edu.cn (Y.-Q.L.); 2 Department of Electronic Engineering, School of Information Science and Engineering, Xiamen University, Xiamen 361005, China; lujianchen@xmu.edu.cn; 3 Department of Chemistry, Tsinghua University, Beijing 100084, China; liangxiao@tsinghua.edu.cn

**Keywords:** cholesteric liquid crystal, photoalignment, light-driven, isomerization, beam steering, micromanipulation, self-assembly, semi-free film

## Abstract

Cholesteric liquid crystal (CLC) has attracted intensive attention due to its ability to form a periodic helical structure with broad tunability. CLC gratings in open systems are especially promising in sensing and micromanipulation. However, there is still much to learn about the inherent mechanism of such gratings. We investigate the light-driven rotation and pitch-tuning behaviors of CLC gratings in semi-free films which are formed by spin-coating the CLC mixtures onto planarly photoaligned substrates. The doped azobenzene chiral molecular switch supplies great flexibility to realize the continuous grating rotation. The maximum continuous rotational angle reaches 987.8°. Moreover, dependencies of light-driven rotation and pitch tuning on the dopant concentration and exposure are studied. The model of director configuration in the semi-free film is constructed. Precise beam steering and synchronous micromanipulation are also demonstrated. Our work may provide new opportunities for the CLC grating in applications of beam steering, micromanipulation, and sensing.

## 1. Introduction

**Figure 1:**
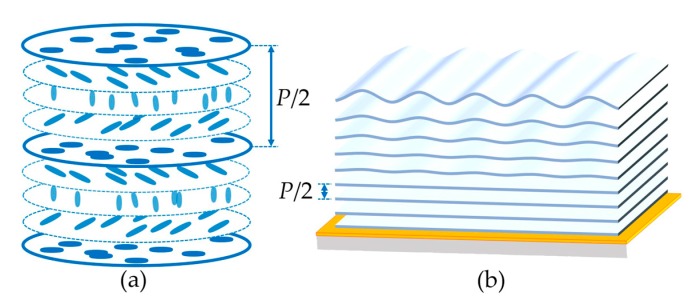
Schematics of (**a**) the cholesteric liquid crystal (CLC) helical structure and (**b**) the helical layers with a self-adapted distortion in a semi-free film. The distance between two adjacent layers equals to half a pitch. The orange layer represents the planar alignment film.

The intrinsic self-assembly of helical structure endows cholesteric liquid crystals (CLCs) the ability to form periodic structure with broadly tunable feature size. The generation of the CLC grating is strongly dependent on the anchoring condition. There are three different modes. In the homogeneous cell [1,2,3,4,5,6], which is composed of two substrates with parallel alignment, the LC molecules spontaneously organize into planar helical layers with the helical axis perpendicular to the substrates. As illustrated in Figure 1a, the helical pitch *P* is defined as the length of one turn of the helical structure. When a proper electric field is applied, the helical axis turns over and subsequently a CLC grating is resulted (Figure S1a). CLC gratings can also be formed in homeotropic cells [7,8,9,10], which is assembled by two vertically aligned substrates (Figure S1b). For the hybrid alignment cell with one substrate planarly aligned and the other one vertically aligned [11,12,13,14,15], helical layers are periodically distorted near the vertically aligned substrate, while layers adjacent to the opposite substrate maintain planar. Thereby, the CLC grating is generated due to the periodic distortion (Figure S1c). All these CLC gratings have merits of easy fabrication, cost efficiency, and high sensitivity to external stimuli [1,2,16,17,18,19], which makes them promising in wide applications, such as diffraction grating [20], non-mechanical beam steering [1,21], polarization-dependent lithography [22], etc. Recently, CLC gratings in semi-free films which break the spatial confinement have attracted intensive attention [23,24] due to their superiorities in sensors and micro-manipulations.

By spin-coating a CLC mixture on a unidirectionally-aligned substrate, a semi-free film is obtained. In this case, the substrate provides a planar anchoring and the air induces a near-vertical anchoring [25]. Therefore, the layered structure of CLC is quite similar to that in hybrid alignment cells. As revealed in Figure 1b, planar helical layers occupy the bulk of the film near the substrate, while periodic distorted helical layers are produced in the vicinity of the LC/air interface, resulting in the CLC grating. Due to the unconstraint of the semi-free film, a regular surface relief emerges [26]. This open system gives rise to novel fantastic applications, such as nanoparticles structuring [27], micro-object manipulation [23,24,28], localized surface plasmon control [25], and so on. Until now, only a few works have focused on this type of CLC grating [23,24,25,26,28,29]. Scientists hold divergent views on the mechanism of the CLC grating. The authors in Refs. [26,28] think that the molecules near the LC/air interface self-assemble into lying helixes, while the others [25,29] consider that the director configuration is similar to that under the hybrid anchoring condition and borrow Baudry’s model [12] to explain the formation of CLC gratings. Therefore, it is an urgent task to systematically study the semi-free CLC materials to uncover hidden rules of the photoinduced rotation behavior and gain insights into the inherent mechanism.

Here, CLC gratings are formed by spin-coating CLC mixtures, doped with a left-handed azobenzene chiral molecular switch onto planarly photoaligned substrates. Due to the photoinduced isomerization of the chiral molecular switch, the helical twisted power (HTP) of CLC can be continuously modified, resulting in the change of layer configuration inside the semi-free film. We investigate the dependencies of light-driven rotation and pitch tuning behaviors of CLC gratings on the dopant concentration and the exposure, respectively. The director configuration in the semi-free film is simulated. On the basis of the new understanding of themechanism and rules of the CLC grating, applications including beam steering and micromanipulation with distinctive characteristics are demonstrated. This work may supply new insights into the promising semi-free material system and inspires more fantastic applications.

## 2. Materials and Methods

### 2.1. Matearials

Photoalignment avoids any mechanical damage, electrostatic charge, and dust contamination; thus, it is suitable for high-quality LC alignment [30]. Here, a polarization-sensitive medium sulfonic azo dye SD1 is utilized as the photoalignment agent. When a linearly-polarized UV or blue light is incident onto the alignment layer, SD1 molecules tend to reorient their long axes perpendicular to the polarization direction in order to minimize the photon absorption, consequently guiding the LC orientation. The resultant azimuthal anchoring energy of the photoalignment film is over 10^−4^ J/m^−2^, which is comparable to that of rubbed polyimide film [31,32,33].

A left-handed azobenzene chiral molecular switch of ChAD-3c-S (BEAM) is adopted for phototuning of the CLC helical structure. It features two azo linkages, which can isomerize from the rod-like *trans*-form to bent *cis*-form structures upon blue light irradiation. The *cis*-form can be stable for several days, while heating or green light exposure will accelerate the *cis*-to-*trans* isomerization [34,35,36]. The influence of the isomerization on helical structures can be quantified by *HTP*,

(1) $$HTP=\frac{1}{P\cdot C\%\cdot ee},$$

where *C* % and *ee* are the concentration and the conformational change of the chiral molecular switch, respectively [26]. The CHAD-3c-S is uniformly dispersed into the achiral nematic LC E7 (HCCH).

### 2.2. Methods

Glass substrates (1.5 × 2 cm^2^) were ultrasonically bathed, UV-ozone cleaned, and then spin coated with SD1. After curing at 100 °C for 10 min, the substrates were photoaligned by a linearly polarized UV light to achieve unidirectional alignment. Then, the ChAd-3c-S-doped CLC mixture was spread onto the substrate at 50 °C and spin-coated at 1000 rpm for 30 s; thus, the semi-free film was obtained. All samples were fabricated under the same condition.

The semi-free films were observed under a transmission polarized optical microscope (POM) (Nikon Eclips 50i Pol, Tokyo, Japan) with a weak light intensity. The photostimulating process was performed under the reflection mode of POM through a bandpass filter (450–490 nm, 229 μW/cm^2^). The diffraction patterns were captured by digital camera (Canon EOS M, Tokyo, Japan).

## 3. Results and Discussion

### 3.1. Light-Driven Rotation and Pitch Tuning Behaviours

**Figure 2:**
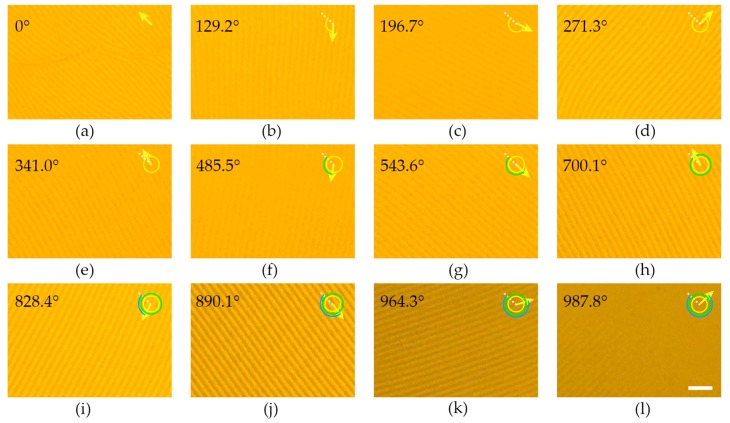
Light-driven rotation of the CLC grating (1 wt % ChAD-3c-S) along with the increasing exposure. The rotational angle is labeled in each top-left corner. The scale bar represents 25 μm for all images.

After the CLC mixture with 1 wt % ChAD-3c-S was spin coated, the CLC grating (initial state) was obtained in a thin semi-free film (Figure 2a). Such CLC films and developed gratings are stable for several days at room temperature under atmospheric condition, but they still suffer from fluidity and dust contamination. The equilibrium helical pitch (*P*_i_) is ~2.3 μm (i.e., 1/*P*_i_ ~0.43 μm^−1^), which is measured by the traditional Grandjean–Cano method [37]. The grating pitch (*Λ*_i_) was measured as ~5.2 μm. Under the irradiation of blue light, the chiral molecular switch isomerizes, modifying the *HTP* and subsequently changing the layer configuration. As presented in Figure 2a–l, along with the exposure increasing, the CLC grating rotates anticlockwise and eventually fades out (end state). The initial and the end grating orientations (*ϕ*) are 135° and 1122.8° (relative to the horizontal axis, the same direction of alignment), respectively. That means that the total continuous rotational angle *α*_t_ of 987.8° was achieved, which is larger than any reported results [11]. Reverse rotation processes can be accomplished by heating or green light exposure. The rotational direction can be switched between clockwise and anticlockwise by changing the incident light (green and blue light, Video S1). The rotational speed is dependent on both the light intensity and the dopant concentration [38]. In the wavelength range of 450–490 nm, the absorbance of SD1 is quite low. And, SD1 molecules are much less sensitive to unpolarized light compared to linearly-polarized light. Therefore, in our experiment, a relatively weak and unpolarized blue light was chosen for both acquiring the details of the grating rotation and guaranteeing the quality of alignment.

**Figure 3:**
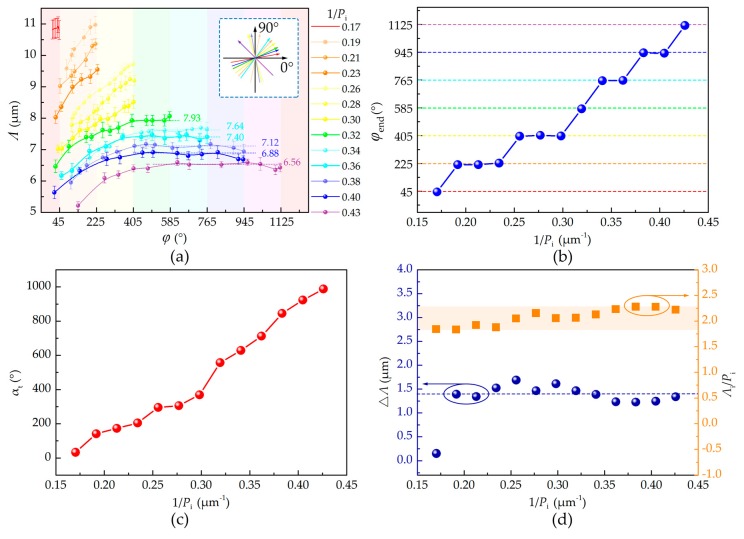
Dependencies of (**a**) *Λ* and *ϕ* on *1/P*_i_, (**b**) $\varphi_{\mathrm{end}}$ on *1/P*_i_, (**c**) *α*_t_ on *1/P*_i_, and (**d**) *△Λ* and *Λ*_i_*/P*_i_ on 1/*P*_i_.

Figure 3a shows the dependencies of the grating pitch *Λ* and *ϕ* on the dopant concentration (labeled by *1/P*_i_). The initial orientation *ϕ*_i_ was measured once the CLC grating is formed, which is in the range of 0° to 180° as depicted in the inset of Figure 3a. The end orientation *ϕ*_end_ was determined when the grating was disappearing (Figure S2). The total continuous rotational angle is defined as *α*_t_ = *ϕ*_end_ − *ϕ*_i_. Other values of *ϕ* (Figure 3a) were selected during the exposure process. Because the contrast of CLC gratings with small *1/P*_i_ is too low to determine the *Λ*, we present the experimental results from *1/P*_i_ = 0.17 μm^−1^. On the basis of the above data, several rules can be concluded. First, as expected, larger 1/*P*_i_ induces smaller *Λ*. Second, *ϕ*_i_ is random, while *ϕ*_end_ = (1/4 + n)π (n = 0, 1, 2, …,6), which is clearly exhibited in Figure 3b. Third, *α*_t_ is directly proportional to 1/*P*_i_ (Figure 3c). Fourth, when 0.17 μm^−1^ ≤ 1/*P*_i_ ≤ 0.30 μm^−1^, *α*_t_ is smaller than 9π/4 and *Λ* monotonically increases with the exposure; when 0.32 μm^−1^ ≤ 1/*P*_i_ ≤ 0.43 μm^−1^, *α*_t_ is larger than 9π/4 and *Λ* will reach and maintain at the maximum *Λ*_max_. The grating pitch is determined by the synergy of elastic force, chiral strength, and anchoring energy. We assume that the anchoring energy is constant. When 1/*P*_i_ is larger than 0.32, although *P* keeps increasing, the influences of ChAD-3c-S isomerization on the elastic force and the chiral strength become balanced. This results in the grating rotation and the maintenance of *Λ*_max_. Fifth, as revealed in Figure 3d, the ranges of the grating pitch tuning *△Λ* are all around 1.38 μm, except for *△Λ* = 0.15 at 1/*P*_i_ = 0.17 μm^−1^ (may be because of a very short time irradiation of blue light). Sixth, the value of *Λ*_i_*/P*_i_ is in the range of 1.8 to 2.3 (Figure 3d), which is smaller than that of a hybrid alignment cell [12]. This phenomenon is attributed to the weaker anchoring at the LC/air interface compared to the homeotropic alignment layer.

### 3.2. Modelling of Director Configuration

Before constructing the model of the director configuration, we analyze some facts in our experiments. The thicknesses *D* of all the samples are constant as they are prepared in the same spin-coating process. Although *P*_i_ is different due to the varied chiral dopant concentrations, the helical pitch at the end state (*P*_end_) should be the same. In this case, *D* is equal to a*P*_end_ for all the semi-free films, where a is a constant describing the residual amount of the helical pitch. This assumption could be verified by the same ends of the cholesteric helixes (director at one end follows the preset alignment and at the other end is reflected by the end orientation of the CLC grating (Figure 3b and Figure S2). We divide the total semi-free film into two different parts. One is composed of planar helical layers, and the other is periodically distorted layers adjacent to the LC/air interface, due to the antagonistic anchoring condition. The CLC grating mainly arises from the phase retardation of the periodic change of LC tilt angle along the grating vector, and the periodic direction is determined by the orientation of a particular planar helical layer. Due to the continuous decrease of *HTP* under the blue light exposure, the cholesteric helixes will unwind gradually, accompanied by the rotation of the grating vector in the same direction.

**Figure 4:**
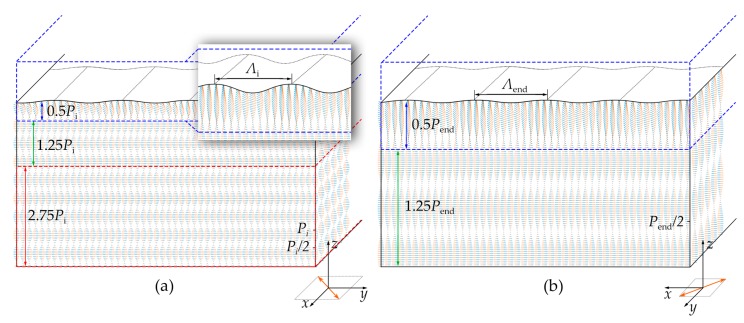
Simulated director configurations of the CLC grating (1 wt % ChAD-3c-S) at the (**a**) initial state and (**b**) end state. The inset in (**a**) shows a zoomed-in image. The anchoring directions are represented by orange arrows.

We simulate the director configuration of the semi-free film based on Baudry’s model [12]. According to the model, periodic distorted helical layers corresponds to 0.5*P*. Here, the initial and end director configurations of the sample (1/*P*_i_ = 0.43 μm^−1^) are constructed (Figure 4). The semi-free film thickness is set as *D* = 4.5*P*_i_0.43_ (Supplementary Material 1), and other required parameters are also determined according to the calculation (Supplementary Material 2). Figure 4a vividly reveals the director configuration of the sample at the initial state. There are four pitches in the planar helical layers. A zoomed-in image of the distorted helical layers (0.5 pitch) is presented as an inset. Figure 4b exhibits the director configuration of the same sample at the end state. Compared with director configuration in Figure 4a, 2.75 pitches are unwound, which is consistent with *α*_t_0.43_/360°. There are still 1.75 residual pitches left at the end state.

### 3.3. Two-Dimentional Precise Beam Steering

**Figure 5:**
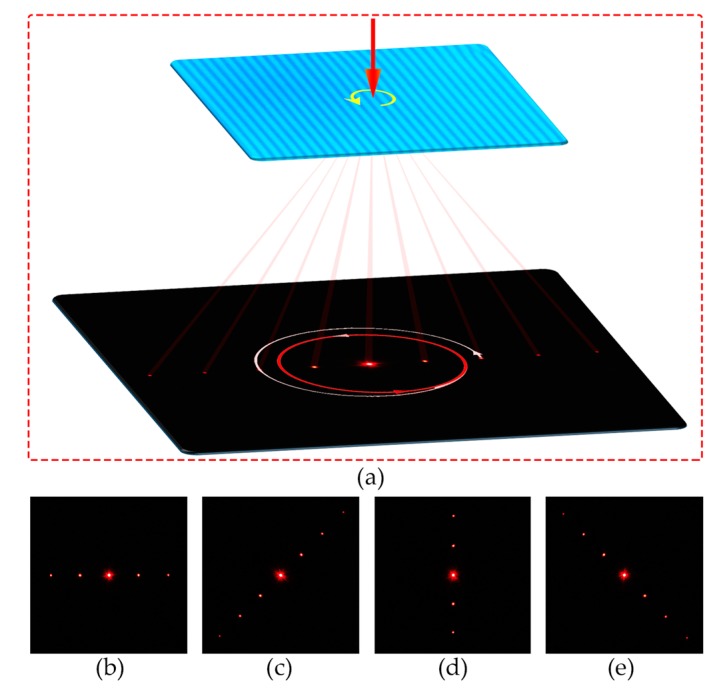
(**a**) Schematic diagram of the optical path for the beam steering characterization. (**b**–**e**) Diffraction patterns collected in the directions of 0°, 45°, 90°, and 135°, respectively.

A 633 nm probe beam is normally incident to the sample of 1/*P*_i_ = 0.43 μm^−1^ to characterize the beam steering performance of the CLC grating. Circular polarization is adopted to avoid the influence of polarization-dependent effect on the diffraction efficiency. Figure 5a schematically shows the optical path for the characterization. The spiral line on the screen depicts the rotational trace of the 2nd diffraction order. At the beginning, the diffraction orders rotate and simultaneously move towards the central 0th order. Afterwards, they would rotate on several fixed concentric circumferences. Four typical photos are selected and exhibited in Figure 5b–e. The above phenomena agree with the results shown in Figure 3a. This facilitates a freely two-dimensional beam steering. Particularly, the rotation of diffraction orders with fixed radius is reported for the first time. It supplies a way for the precise control of beam propagation in a photoresponsive manner.

### 3.4. Synchronous Microparticle Rotation

**Figure 6:**
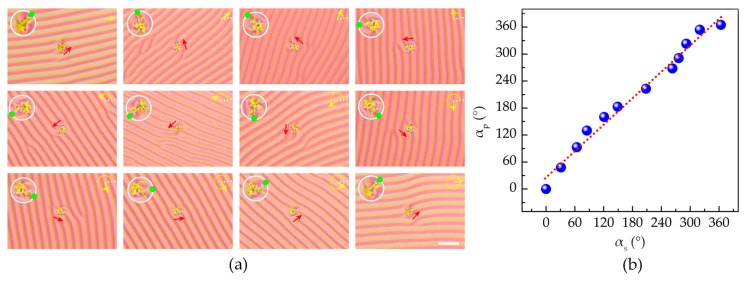
(**a**) Polarized optical microscope textures of the ZSM-5 zeolite microparticle rotation on the semi-free film. The enlarged microparticle is presented in the top left corner with a green dot denoting its orientation. The scale bar represents 25 µm for all images. (**b**) Relationship between the rotational angle of the microparticle and that of the CLC grating. The dashed line is the linear fitting result.

ZSM-5 zeolite microparticles are chosen for the demonstration of micromanipulation. The typical length of the long axis is ~12.6 µm and the aspect ratio is ~1.32. The microparticles of ZSM-5 zeolite are spattered onto the surface of the semi-free film, and then their rotation behaviors are monitored. Figure 6a shows the rotation of one of the microparticles. It rotates anticlockwise along with the rotation of the grating. The relationship between the rotational angle of microparticle and that of the CLC grating is depicted in Figure 6b. The fitted slope is ~0.98, suggesting that the particle could be precisely manipulated. Note that, after the grating disappears, the ZSM-5 zeolite can rotate with an extra angle of ~430°, which verifies that residual cholesteric helixes are still left at the end state. This finding opens a door for the precise micromanipulation by utilizing the cholesteric semi-free material system.

## 4. Conclusions

In summary, we investigate the light-driven rotation and pitch tuning of CLC gratings in semi-free films. Due to the continuous photoinduced modification of *HTP*, the CLC grating rotates along with the unwinding cholesteric helixes. This supplies great flexibility to the realization of continuous grating rotation. Dependencies of the light-driven rotation and pitch tuning on the dopant concentration and exposure are studied. The maximum rotational angle reaches 987.8°. The end grating orientations of all samples are fixed at the same direction. On the basis of the new understanding on the mechanism and rules of CLC gratings, the layer configuration of the semi-free film is simulated. The model depicts the director configuration of the sample and vividly reveals the relationship between the helixes unwinding and grating rotation. Distinct superiorities of the CLC gratings and applications including beam steering and micromanipulation are also demonstrated. Phenomena such as diffraction orders rotation on fixed concentric circumferences and synchronous rotation of microparticles with the CLC grating are reported for the first time. This work may offer new insights into the promising semi-free structured materials and inspire more fantastic applications.

## Supplementary Materials

The following are available online at www.mdpi.com/2073-4360/9/7/295/s1, Figure S1: Schematic illustrations of the CLC grating in cells under the homogeneous, homeotropic and hybrid anchoring conditions. Figure S2: Polarized optical microscope textures of the end states of CLC gratings. Video S1: Reverse rotation processes of the CLC grating by switching the blue and green light. Supplementary Material 1: Determination of the semi-free film thickness. Supplementary Material 2: Modeling of director configuration of the CLC grating in a semi-free film.
