# Supplementary material for: Light-Driven Rotation and Pitch Tuning of Self-Organized Cholesteric Gratings Formed in a Semi-Free Film
Source: Polymers (Basel). 2017 Jul 21;9(7):295. doi: 10.3390/polym9070295 (PMC6432147; doi:10.3390/polym9070295)
Supplement: Supplementary file 1 [file polymers-09-00295-s001.zip › polymers-210293-Supplementary.pdf]

Supplementary Materials

Supplementary Material Figures:

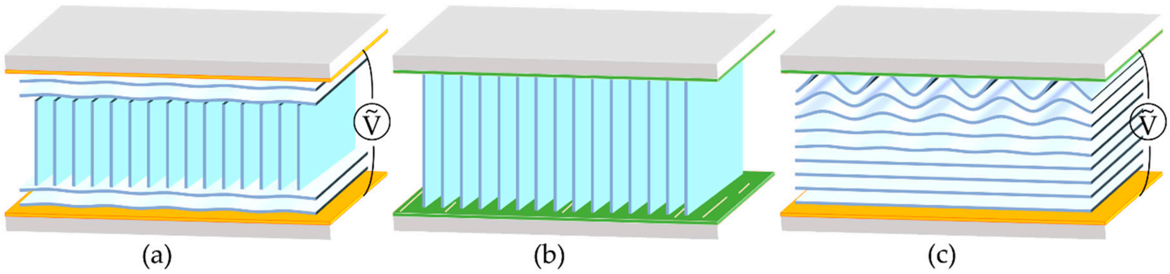

Figure S1. Schematic illustrations of the CLC grating in cells under the (**a**) homogeneous, (**b**) homeotropic and (**c**) hybrid anchoring conditions. The orange and green layers represent the planar and vertical alignment layers respectively, and the blue ones represent the cholesteric helical layers.

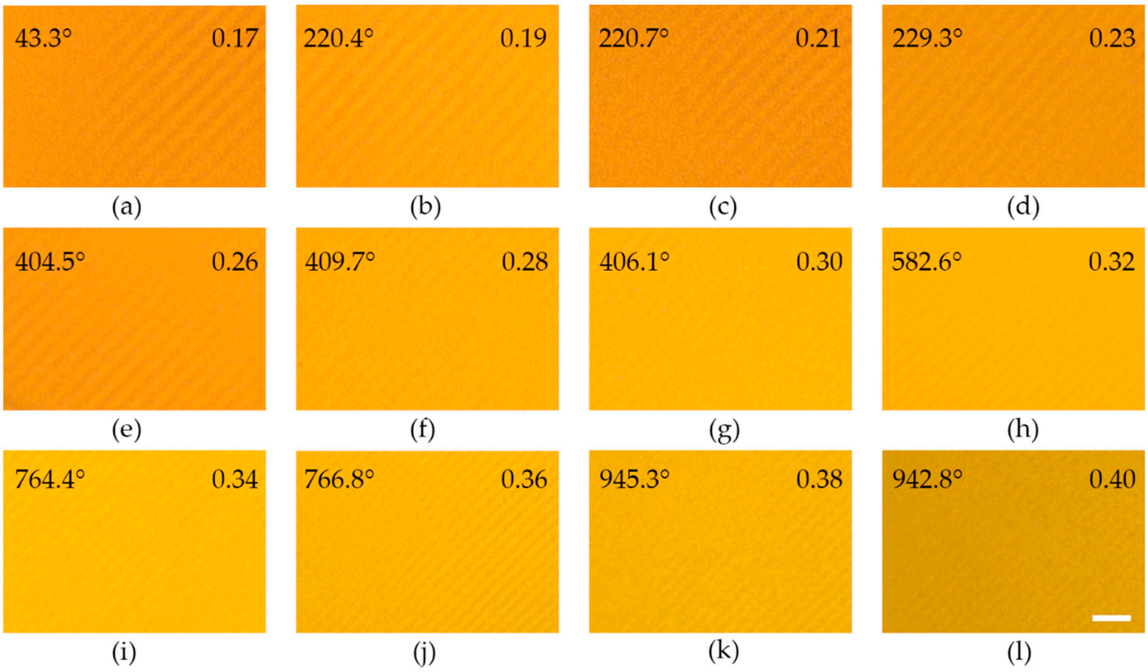

Figure S2. Polarized optical microscope textures of end states of CLC gratings. φ_{end} and $1/P_i$ are denoted on the top left and top right corners, respectively.

Supplementary Material Video:

Video S1: Reverse rotation processes of the CLC grating by switching the blue and green light. The grating period is 6.6 μm. The speed variation is ×9. The intensity of blue light (450~490 nm) is 229 μW/cm^2 and the intensity of Green light (510~560 nm) is 338 μW/cm^2.

Supplementary Material 1: Determination of the Semi-Free Film Thickness

On the basis of the experimental results $\alpha_{t\_0.17} = 33.0°$, $P_{i\_0.17} = 5.9$ μm, when $1/P_i$ is 0.17 μm^{-1}, and $\alpha_{t\_0.43} = 987.8°$, $P_{i\_0.43} = 2.3$ μm, when $1/P_i$ is 0.43 μm^{-1}, we can get: $d_{3\_0.17} = \frac{\alpha_{t\_0.17}}{360} \cdot P_{i\_0.17} = 0.09P_{i\_0.17}$ μm and $d_{3\_0.43} = \frac{\alpha_{t\_0.43}}{360} \cdot P_{i\_0.43} = 2.74P_{i\_0.43}$ μm. Because of $d_1 = 0.5P_i$, simultaneous equations are constructed:

$$D_{0.17} = d_{1\_0.17} + d_{2\_0.17} + d_{3\_0.17} = 0.5P_{i\_0.17} + \mathrm{a} \cdot P_{i\_0.17} + 0.09P_{i\_0.17}, \quad (1)$$

$$D_{0.43} = d_{1\_0.43} + d_{2\_0.43} + d_{3\_0.43} = 0.5P_{i\_0.43} + \mathrm{a} \cdot P_{i\_0.43} + 2.74P_{i\_0.43}, \quad (2)$$

$$D_{0.43} = D_{0.17}, \tag{3}$$

After calculation, we get $a = 1.14$, $D_{0.17} = 1.73 P_{i\_0.17}$, $D_{0.43} = 4.38 P_{i\_0.43}$. In our simulation, we take $d_1 = 0.5 \cdot P_{i\_0.43}$, $d_2 = 1.25 \cdot P_{i\_0.43}$ and $d_3 = 2.75 \cdot P_{i\_0.43}$ to simplify the modelling.

Supplementary Material 2: Modeling of Director Configuration

We assign z-axis perpendicular to the substrate, x-axis deviating 45° with respect to the anchoring direction. The anchoring direction is at $\varphi = \pi/4$, along the orange arrow. According to the Baudry's model [1], the director field in the semi-free film is:

$$n_x = [\cos(\theta+\gamma)\cos\phi\cos\rho - \sin\phi\sin\rho]\sin\gamma + \sin(\theta+\gamma)\cos\phi\cos\gamma, \tag{4}$$

$$n_y = [\cos(\theta+\gamma)\sin\phi\cos\rho + \cos\phi\sin\rho]\sin\gamma + \sin(\theta+\gamma)\sin\phi\cos\gamma, \tag{5}$$

$$n_z = -\sin(\theta+\gamma)\cos\rho\sin\gamma + \cos(\theta+\gamma)\cos\gamma, \tag{6}$$

where $\theta(z)$ is the tilt angle of the director: $\theta(z) = \frac{\pi}{2}$ for $z \in [0, D-d_1]$ and $\theta(z) = \frac{\pi}{2d_1}(D-z)$ for $z \in [D-d_1, D]$. $\phi(z)$ is the angle between the grating vector and the anchoring direction: $\phi(z) = \frac{2\pi}{P_e} \cdot z + \frac{\pi}{4}$, $P_e = 2.3$ μm^{-1}. To balance the surface energy and the elastic energy, $\gamma(z)$ is introduced to represent the transversally conic propagation along the grating vector: $\gamma(z) = 0$ for $z \in [0, D-d_1]$, $\gamma(z) = \frac{2\gamma_0}{d_1}(z - D + d_1)$ for $z \in [D-d_1, D-\frac{d_1}{2}]$ and $\gamma(z) = \frac{2\gamma_0}{d_1}(D-z)$ for $z \in \left[D-\frac{d_1}{2}, D\right]$. γ_0 is the half apex angle of the cone, which is assigned the value of 0.3 rad [1]. $\rho(z)$ is the real-time conic propagation parameter: $\rho(z) = \frac{2\pi}{\Lambda_i} \cdot y$. $\Lambda_i = 5.2$ μm, $\Lambda_{\text{end}} = 6.6$ μm.

1. Baudry, J.; Brazovskaia, M.; Lejcek, L.; Oswald, P.; Pirkl, S. Arch-texture in cholesteric liquid crystals. *Liq. Cryst.* **1996**, *21*, 893-901, 10.1080/02678299608032907.
